# A Pilot Study of the Role of Selected Biomarkers of Kidney Injury in Dogs with Dilated Cardiomyopathy

**DOI:** 10.3390/ani14091305

**Published:** 2024-04-26

**Authors:** Karolina Wrześniewska, Jacek Madany, Dawid Tobolski, Beata Żylińska, Andrzej Milczak, Aleksandra Sobczyńska-Rak

**Affiliations:** 1 Department and Clinic of Animal Internal Diseases, Faculty of Veterinary Medicine, University of Life Sciences in Lublin, 13 Akademicka Street, 20-950 Lublin, Poland; karolina.wrzesniewska@up.lublin.pl (K.W.); jacek.madany@up.lublin.pl (J.M.); andrzej.milczak@up.lublin.pl (A.M.); 2 Department of Animal Reproduction with Clinic, Faculty of Veterinary Medicine, University of Warmia and Mazury, 14 Oczapowskiego Street, 10-719 Olsztyn, Poland; dawid.tobolski@uwm.edu.pl; 3 Department and Clinic of Animal Surgery, Faculty of Veterinary Medicine, University of Life Sciences in Lublin, 13 Akademicka Street, 20-950 Lublin, Poland; olsob2@gmail.com

**Keywords:** dilated cardiomyopathy in dogs, early renal dysfunction detection, canine cardio–renal axis disorders, biomarkers in veterinary nephrology, NGAL, cystatin C, KIM-1

## Abstract

**Simple Summary:**

The concept of the cardio–renal axis is based on the assumption that kidney dysfunction is a secondary consequence of heart disease, a mechanism observed in dilated cardiomyopathy (DCM). Traditional serum markers are not always effective, especially in the early stages of kidney disease. Thus, there is a need to find more sensitive measures of renal impairment during heart failure. The selected biomarkers, cystatin C, KIM-1 (kidney injury molecule-1), and NGAL (neutrophil gelatinase-associated lipocalin), were evaluated in a study for their efficacy as indicators of early kidney damage in dogs with dilated cardiomyopathy. The serum concentrations of these biomarkers and their creatinine ratio were measured and analyzed to determine their diagnostic values. This study’s findings suggest that during the asymptomatic phase of dilated cardiomyopathy, only NGAL concentration and the NGAL/creatinine ratio may serve as diagnostic markers for early-stage kidney injury. The above markers can, therefore, be used in the early diagnosis of kidney diseases in dogs with asymptomatic DCM.

**Abstract:**

Heart and kidney diseases are among the most frequent medical conditions diagnosed in small animals. Due to the functional interconnection between these organs, the concept of the cardio–renal axis has been developed. In this context, renal disease or dysfunction often occurs secondary to heart diseases, such as dilated cardiomyopathy (DCM). DCM is the most common cardiomyopathy and a leading cause of mortality in large-breed dogs. Traditional biomarkers like creatinine or symmetric dimethylarginine concentration are not always effective, especially in the early stages of the disease, underscoring the need for more sensitive markers of renal impairment during heart failure (HF). This study aimed to evaluate the efficacy of selected biomarkers as indicators for early kidney damage in dogs with stage B2 DCM. We measured serum concentrations of cystatin C, KIM-1 (kidney injury molecule-1), and NGAL (neutrophil gelatinase-associated lipocalin) and their ratios to creatinine, analyzing their diagnostic values. Cystatin C was quantified using a sandwich enzyme immunoassay, while KIM-1 and NGAL were measured with enzyme-linked immunosorbent assay kits designed for canine diagnostics. The concentrations were indexed against serum creatinine. The study included 26 dogs: 9 with HF and 17 healthy controls. The mean ± standard deviation for healthy dogs for cystatin C, cystatin C/creatinine ratio, KIM-1, KIM-1/creatinine ratio, NGAL, and NGAL/creatinine ratio were 0.24 ± 0.04, 0.26 ± 0.07, 0.61 ± 0.07, 0.67 ± 0.13, 2.76 ± 1.8, and 2.79 ± 1.81, respectively. For DCM dogs, these values were 0.27 ± 0.1, 0.32 ± 0.12, 0.61 ± 0.08, 0.69 ± 0.17, 6.46 ± 5.22 (*p* = 0.02), and 7.99 ± 6.53 (*p* = 0.04). This study’s findings suggest that during the asymptomatic phase of DCM, only NGAL concentration and the NGAL/creatinine ratio may serve as diagnostic markers for early-stage kidney injury.

## 1. Introduction

Heart and kidney diseases are the leading causes of mortality in small animals, significantly affecting both life expectancy and quality of life [1]. In the realm of canine cardiac disorders, cardiomyopathies are a frequent cause of cardiac disability, congestive heart failure (CHF), and arrhythmic death in dogs [2]. Among cardiomyopathies, primary dilated cardiomyopathy (PDCM) is the most prevalent form in mid-sized and large-breed dogs [3,4,5]. The condition impairs both mechanical and electrical functions of the heart and is often genetic in nature. To diagnose an intrinsic cardiomyopathy, known as primary dilated cardiomyopathy, it is essential to rule out “extramyocardial” disorders (like ischemia; valvopathies; cardiac arrhythmias; systemic arterial hypertension; and immune-mediated, nutritional, infectious, toxic, endocrine, or neuromuscular diseases) [3,5,6,7]. Its significance lies in being a major cause of CHF and sudden cardiac death in these dog breeds. The genetic underpinnings of idiopathic DCM are complex and believed to involve multiple genes, which complicates its diagnosis and management. A comprehensive understanding of idiopathic DCM, encompassing its prevalence and impact, is vital for enhancing veterinary care and improving the quality of life of the affected dogs [4,6,7].

In some dog breeds, DCM is frequently associated with arrhythmias, notably atrial fibrillation (AF) and ventricular premature complexes (VPCs). This complicates the clinical manifestation of DCM [7]. The currently recommended staging system modified for canine DCM is divided into four stages: A, B (subdivided into stages B1 and B2), C, and D. Stage A is specified by the absence of any cardiac disease visible on echocardiography or Holter monitoring. This stage includes predisposed breeds and dogs tested positive for a known genetic mutation but without detectable manifestations of DCM. Stage B1 is characterized by the absence of morphological changes but includes dogs exhibiting ventricular or supraventricular arrhythmias due to DCM. Conditions such as VPCs in Doberman Pinschers or Boxers and AF in Irish Wolfhounds or giant breeds are common at this stage. Stage B2 is identified in dogs with left ventricle systolic dysfunction, with or without concurrent left ventricle chamber enlargement in systole and diastole. Arrhythmias may also be present at this stage. Stage C encompasses dogs exhibiting current or prior clinical manifestations of congestive heart failure (CHF), often accompanied by severe cardiac remodeling. Stage D refers to dogs with refractory CHF [7].

As described by Bulmer, heart disease in dogs often progresses [8]. Initially, the progression is mild and slow, with early heart damage often presenting no detectable clinical signs, making this phase particularly insidious. A progressive decrease in myocytes and a decline in myocyte function cause myocardial thinning and a decrease in contractility. These pathophysiological changes include a decrease in blood volume pumped by the expending ventricle at each contraction and the amount of blood pumped by the heart per minute, impaired ventricular filling, and an increase in end-diastolic pressure [9]. As the condition advances to the second phase, the body engages short-term and long-term compensatory mechanisms to stabilize cardiac output and normalize afterload. The most well-known compensatory homeostatic responses to a decrease in cardiac output are the sympathetic nervous system (SNS) and the renin–angiotensin–aldosterone system (RAAS). Increased SNS activity enhances contractility, heart rate, the rate of ventricular relaxation, and the speed of impulse conduction through the heart and induces arterial and venous vasoconstriction. The RAAS complements the SNS by maintaining blood pressure through increased salt and water retention. However, these are not long-term solutions and only temporarily support heart function. The third phase is marked by the onset of clinical signs such as exercise intolerance, lethargy, coughing, and tachypnea. At this stage, the heart struggles to maintain adequate cardiac output, despite eccentric hypertrophy, due to the chronic and progressive nature of cardiovascular disease. The compensatory mechanisms that initially provided stability may become counterproductive [8]. Sustained neurohormonal activation leads to maladaptive extracellular matrix remodeling, lengthening of individual myocytes, and LV dilation. Such physiological alterations negatively impact the heart’s ability to contract effectively. The culmination of these alterations in the myocardium and cardiac function ultimately leads to the manifestation of heart failure and arrhythmias [10]. This progression underscores the critical importance of early detection and management to improve the quality of life and prolong the longevity of affected dogs.

As heart disease evolves, dogs may develop systolic and/or diastolic dysfunction, leading to failure seen as venous stasis in either systemic or pulmonary circulation due to increased venous pressure. Detecting these stages promptly enables the timely initiation of both prophylactic measures and pharmacological treatments, essential for delaying the onset of improving the quality of life and extending the survival of dogs suffering from heart failure [8,11,12,13].

Kidney damage in small animals can manifest as acute kidney injury (AKI) or, more commonly, chronic kidney disease (CKD), often as a consequence of heart-related processes. The co-occurrence of conditions like CHF and AKI/CKD significantly compounds the clinical challenges. This intersection underscores the importance of “advance diagnosis”—the identification of changes in both the heart and kidneys at an asymptomatic stage. Consequently, recognizing early cardiac and renal changes, even before clinical symptoms manifest, is crucial [14,15].

Cardiology has made significant advances in diagnostics, including echocardiography and Holter examinations. However, nephrology faces challenges in the early-stage diagnosis of kidney damage. Traditional methods, like measuring creatinine concentration as recommended by the International Renal Interest Society (IRIS) for staging CKD in dogs and cats, often fail to detect renal dysfunction in its early stages. This diagnostic gap has prompted the search for new biomarkers that can more accurately and promptly detect kidney damage [16].

Cardiorenal syndrome (CRS) encompasses a range of complex conditions involving both the heart and kidneys, where dysfunction in one organ can precipitate or exacerbate dysfunction in the other. [17]. This syndrome encapsulates multiple subtypes based on the pathology and chronicity of the condition: Type 1 (acute cardiorenal), involving acute worsening of heart function leading to kidney injury; Type 2 (chronic cardiorenal), where chronic heart disease leads to kidney injury; Type 3 (acute nephrocardiac), characterized by acute worsening of kidney function causing heart injury; Type 4 (chronic nephrocardiac), where chronic kidney disease leads to heart injury; and Type 5, involving systemic diseases that cause both heart and kidney failure [17,18]. The cardiovascular renal axis disorder (CvRD) concept is pivotal in understanding the intricate interplay between heart and kidney diseases in dogs and cats, categorizing CvRD into five subtypes based on the primary causative factor. Cardiovascular renal axis disorder (stable disease) typically results in progressive chronic kidney disease caused by chronic cardiovascular disease (CvRDH) [19,20].

Several studies have examined the progression of chronic kidney disease in dogs with myxomatous mitral valve disease, notably those by Martinelli [21] and Yun [22]. The prevalence and impact of CvRD in dogs with DCM have not been extensively studied. Diagnosing DCM based solely on full clinical signs often proves inadequate, especially considering the lengthy asymptomatic period characteristic of the disease. This underscores the importance of early detection, particularly in the preclinical stage of DCM. While electrocardiogram (ECG) recordings have limited diagnostic value in the early detection of DCM [7,16,23,24], a Holter recording is recommended for screening examination in Doberman Pinschers and might be indicated in other breeds as well [16].

Cystatin C, a low-molecular-weight protein produced by all nucleated cells, is completely filtered and reabsorbed by the kidneys. Notably, serum cystatin C levels are not influenced by age, sex, race, or muscle volume, making it a potential alternative to serum creatinine. Studies in humans and dogs have demonstrated that cystatin C is a more sensitive and specific indicator of kidney dysfunction than serum creatinine [25,26,27,28].

KIM-1, a type I transmembrane glycoprotein, is minimally expressed in normal kidneys, but its expression significantly increases in proximal tubule cells following kidney injury. It has been found that KIM-1 can detect patients with AKI or CKD more quickly than serum creatinine levels [14,15,29,30].

NGAL, a protein in the lipocalin family, is another extensively studied biomarker for the early diagnosis of renal dysfunction in humans. It has been employed as a predictor of AKI, CKD, and the need for renal replacement therapy, even before an increase in serum creatinine is observed [14,31,32,33,34,35,36].

In this research, the selected biomarkers were used to assess two crucial aspects of kidney health: the filtration function of the glomeruli and the integrity of the tubular epithelium. These markers are essential for detecting early renal changes influenced by developing heart disease in dogs. A significant precedent for this approach was established by Koch et al. in [37], who showed that the RAS tends to be activated in dogs with asymptomatic DCM. This discovery underlines the need for early therapeutic intervention in such cases, emphasizing the importance of timely diagnosis and treatment in potentially mitigating the progression of both cardiac and renal complications in dogs with DCM [37]. The primary aim of this study was to evaluate the effectiveness of selected renal dysfunction biomarkers (cystatin C, KIM-1, and NGAL) in dogs with stage B2 DCM compared to a healthy control group. This evaluation focused on assessing nephron damage through serum concentration markers and exploring the diagnostic potential of these biomarkers in the context of DCM. Furthermore, this study sought to gather preliminary data that could inform the sample size calculation for more comprehensive studies in this field in the future.

## 2. Materials and Methods

### Animals and Group Assignment

This study involved 26 dogs referred to the Department and Clinic of Animal Internal Diseases at the Faculty of Veterinary Medicine in Lublin. Their owners were informed about the research and consented to its scope. The animals were brought for preventive breed-specific consultations or routine check-ups. Each dog underwent a thorough medical history review, including signalement, and a comprehensive clinical examination focusing on heart and lung auscultation. This included blood pressure measurement; electrocardiograms (ECGs); thoracic chest radiographs; cardiac echocardiography; and hematological, biochemical, and urinary tests.

Echocardiography was conducted using an ESAOTE MY LAB 40 eHD device with a 1.0–4.0 MHz phased array transducer. Breed-specific criteria were applied when appropriate [22,38,39,40,41,42,43,44]. The examination followed existing guidelines and was performed on an echocardiographic table in right and left lateral recumbency.

The echocardiographic assessment employed a conventional protocol encompassing 2-D, M-mode, spectral Doppler, and color Doppler techniques. This allowed for the classification and staging of dogs with dilated cardiomyopathy (DCM) according to established guidelines [22,38,39,40,41,42,43,44,45]. The dogs exhibited echocardiographic changes indicative of systolic dysfunction, such as increased left ventricular (LV) end-systolic chamber diameter or end-systolic volume, reduced fractional shortening or ejection fraction, and increased E-point to septal separation. Signs of volume overload were also observed, including diastolic LV diameters or volumes exceeding reference ranges [7,14,22,23,37]. Color and spectral Doppler examinations were used to rule out other acquired or congenital cardiac diseases [7].

The inclusion criterion for the study was the reduction of the shortening fraction (SF) and the systolic left ventricular dilation in dogs that had not previously been treated with pharmacological agents. Exclusion criteria encompassed AKI and CKD in the symptomatic phase according to IRIS guidelines, taurine and L-carnitine deficiencies, hypothyroidism, medications, multi-organ failure, infectious diseases, cancer, musculoskeletal diseases, anemia, proteinuria, and autoimmune diseases.

Blood pressure measurements were performed using the Doppler method. This involved using a VetBP-type MD4-CW8 device from SONOMED, Poland, in a quiet setting with the dogs in a standing position. With a recommended cuff width of 30% to 40% of the circumference of the appendage (fore-limb), five measurements were made, two of which were rejected, and the average was taken. For electrocardiogram (ECG) evaluations, a BTL-08SD3 device from Poland was used, with dogs positioned in right lateral recumbency. This procedure included attaching electrode alligator clips to each limb and conducting 6-lead ECGs following the standard guidelines [46].

Thoracic radiographs were carried out using an ARCOMA device, Varian Tube Type RAD-14, USA, equipped with a direct radiography system for digital image analysis. The heart was assessed in lateral and ventrodorsal projections. In the lateral view, heart silhouette position and size were measured using the vertebral heart size (VHS) scale, taking into account breed, size, body condition score (BCS), and clinical examination results [38,47]. In the ventrodorsal projection, anatomical heart and vessel parameters were considered for evaluating heart size. Additionally, the condition of the lungs and the level of air filling were analyzed.

Blood samples for laboratory tests were collected either on the day of the visit or the following day. Hematological and biochemical profiles were assessed, focusing particularly on renal function, using standard diagnostic procedures. Hematological tests used a closed vacuum system with EDTA K2 tubes, with immediate analysis on a HORIBA MEDICAL SCIL VET ABC+ veterinary hematology analyzer from Japan. This provided a complete set of hematological indicators, including white blood cell count and percentages.

Biochemical tests were conducted on serum obtained after centrifuging whole blood at 1000 rpm for 15 min at room temperature. Serum was placed in plain vacutainer tubes and centrifuged at 1000× *g* for 7 min. Analysis was performed using the HORIBA ABX PENTRA 400 biochemical analyzer from Japan, measuring creatinine, urea, total protein, AST, and selected electrolytes (sodium, potassium, and ionized calcium) using the AVL 9180 electrolyte analyzer from Roche, Switzerland.

Additionally, biomarkers indicative of kidney damage were analyzed in the blood serum. The serum that had been collected was stored at a temperature of −70 °C. Once an adequate number of samples had been gathered, the levels of cystatin C, KIM-1, and NGAL were determined through the enzyme-linked immunosorbent assay (ELISA) method. The assays employed were the Canine Cystatin C ELISA by BIOVENDOR, Czech Republic, and the ELISA kit for KIM-1 and the ELISA kit for NGAL, both from CLOUD-CLONE CORPORATION, USA, and specifically for canine species.

The concentration of serum cystatin C was measured using a species-specific sandwich enzyme immunoassay according to the manufacturer’s instructions. The results are reported in mg/L. The kit included a plate with wells that were precoated with a polyclonal anti-canine cystatin C antibody, along with standards and quality controls. The intra- and inter-assay coefficients of variation (CV) for serum cystatin C were recorded as 8.41% (serum 1) and 4.20% (serum 2) and 4.12% (serum 1) and 7.28% (serum 2), respectively. The concentrations of the samples, calculated from the standard curve, were adjusted by their dilution factor (×1000), as the samples had been diluted before the assay. These samples were tested in duplicate, and the necessary dilutions were made using the diluent provided in the kit. One hundred microliters of standards, quality controls, dilution buffer, and samples were placed in the precoated wells. Following 60 min of incubation, five automated washing series were conducted using the washing solution included in the kit. Then, 100 microliters of the conjugate solution were added to each well and incubated at room temperature for another 60 min. After this incubation, another series of washes was performed, and the substrate solution was added for a third incubation of 15 min under aluminum foil. The stop solution was applied after this final incubation.

The concentrations of serum KIM-1 and NGAL were also measured using species-specific enzyme-linked immunosorbent assay kits following the manufacturer’s instructions. The results are expressed in ng/mL. These kits consisted of plates with precoated wells as well as the necessary reagents and standards. For serum KIM-1, the intra- and inter-assay CV were less than 10% and 12%, respectively, while for serum NGAL, these values were also below 10% and 12%, respectively. One hundred microliters of standards and samples, in duplicate, were placed in the precoated wells and incubated for 60 min at 37 °C, followed by aspiration and the addition of 100 microliters of the prepared detection reagent A. After another 60 min of incubation, three automated washing series were conducted using the washing solution. Then, 100 microliters of the prepared detection reagent B were added. After 30 min of incubation at 37 °C, five more washing series were performed before adding the substrate solution for a fourth incubation lasting 20 min at 37 °C. The stop solution was applied after this final incubation. The optical density of the solutions in each well was measured at 450 nm using a SpectraMax M2 multimode plate reader (Molecular Devices, San Jose, CA, USA).

The data were analyzed using the Python (Python Software Foundation, Wilmington, DE, USA, v 2023), R (R Core Team, 2023) programming languages, and the JASP application (JASP Team 2020; JASP, Version 0.14.1). Descriptive statistics are presented as the means and standard deviations. The normality of distributions was determined using the Shapiro–Wilk test. Due to the failure to meet the assumptions of normality, non-parametric tests were used to analyze the variables. A comparison between the study groups was performed using the Mann–Whitney U test. The proportions were compared using the chi-square test. Figures were made using the Matplotlib library.

## 3. Results

The study involved nine dogs diagnosed with DCM, including eight purebreds (a Boxer, a Doberman Pinscher, a German Shepherd, two Golden Retrievers, an Irish Setter, and two Labrador Retrievers) and a German Shepherd mix. This group comprised three females and six males, aged 6–10 years, with weights ranging from 25 to 40 kg. The control group consisted of 17 healthy dogs (7 crossbreeds, 3 English Cocker Spaniels, Border Collie, Maltese, Australian Shepherd, Rottweiler, Staffordshire Bull Terrier, Whippet, and Yorkshire Terrier), including 5 females and 12 males, aged between 3 and 9 years and weighing between 3 and 38 kg. Physical examination findings, routine hematology, biochemical testing, and the sex distribution of the dogs are detailed in Table 1. Notably, none of the dogs were under treatment for heart disease at the time of the study.

Body condition scores in the control group were within the ideal range, while two dogs in the DCM group were slightly overweight (about 10% above their ideal weight). Key health indicators such as body temperature, systolic blood pressure, packed cell volume, white blood cell count, serum total protein, AST, sodium, potassium, and calcium levels were found to be within normal ranges for all dogs. Heart rhythm assessments, including ECG readings, showed normal heart rates without any abnormal heart rhythms being detected.

Interestingly, serum creatinine concentrations were slightly higher in the healthy control group (0.084 mmol/L) compared to the DCM group (0.080 mmol/L). However, all dogs had serum creatinine levels below 0.12 mmol/L, which is within the normal range. Serum urea concentrations were slightly lower in the control group (6.59 mmol/L) than in the DCM group (7.32 mmol/L), but again, all values fell within the reference range. Statistical analysis revealed no significant differences in these parameters between the groups.

A summary of the kidney biomarkers in serum, including cystatin C, KIM-1, and NGAL, and their ratios to creatinine, are shown in Table 2. Significantly higher levels of NGAL and the NGAL/creatinine ratio were observed in the DCM group (6.46 ± 5.22 ng/mL and 7.99 ± 6.53, respectively) compared to the control group (2.76 ± 1.8 ng/mL and 2.79 ± 1.81), with *p*-values of 0.02 and 0.04, respectively.

**Figure 1:**
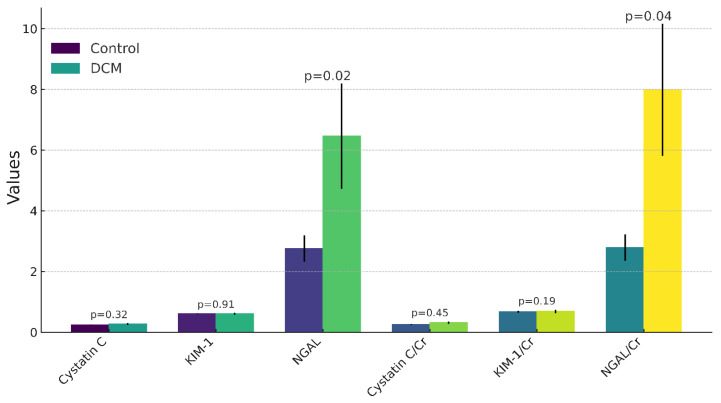
Biomarker comparison between the control and DCM groups. The figure presents a bar graph comparing six biomarkers between a control group (*n* = 17) and a dilated cardiomyopathy (DCM) group (*n* = 9). Biomarkers, including cystatin C, KIM-1, NGAL, and their ratios to creatinine (Cr), are measured in mg/L, ng/mL, and dimensionless units, respectively. Mean values are depicted by bar heights, with the standard error of means (SEMs) indicated by error bars. Significant differences were observed in NGAL (*p* = 0.02) and the NGAL/Cr ratio (*p* = 0.04), suggesting their potential diagnostic significance in DCM. The graph provides a clear visualization of biomarker variations, aiding in the assessment of their clinical relevance in DCM.

In contrast, no significant differences were found between the healthy dogs and those with DCM in terms of cystatin C, cystatin C/creatinine ratio, KIM-1, and KIM-1/creatinine ratio (0.24 ± 0.04 mg/L, 0.26 ± 0.07, 0.61 ± 0.07, 0.67 ± 0.13 vs. 0.27 ± 0.1 mg/L, 0.32 ± 0.12, 0.61 ± 0.08, 0.69 ± 0.17 ng/mL, respectively) (Table 2, Figure 1).

## 4. Discussion

This pilot study represents a pioneering effort in exploring the role of specific biomarkers in detecting early kidney damage in dogs with DCM, particularly in the context of the cardiovascular–renal axis disorders of the heart (CvRDH) subtype. CvRDH specifically addresses the development of kidney disease—either acute kidney injury (AKI) or, more commonly, chronic kidney disease (CKD)—as a secondary consequence of DCM.

In clinical settings, kidney damage is typically identified using various urinary and serum indicators. These include urine specific gravity, urine protein content, and serum concentrations of urea, creatinine, and symmetric dimethylarginine (SDMA). However, these indicators have limitations in sensitivity and specificity. Variations in urine can be influenced by non-renal and metabolic factors, while proteinuria, although a well-established marker of kidney damage, is dependent on several variables. Proteinuria signifies an abnormal presence of protein in urine and is recognized both as a marker for chronic kidney disease severity and a predictor of future renal function as well as cardiovascular morbidity and mortality [48,49,50,51]. Several studies have affirmed proteinuria’s role as an independent risk factor for renal dysfunction in both CKD [52,53,54] and AKI [49,51,55]. Flammia et al. [48] highlighted proteinuria’s significance as a prognostic factor for shorter survival and impaired kidney function in humans [48]. While similar comprehensive studies are not available for dogs, the possibility of a comparable effect in canine patients cannot be ruled out.

Serum urea and creatinine concentrations can be influenced by various factors, such as age, diet, muscle mass, tissue hydration, and liver function [20,56,57,58,59]. These multifactorial influences on serum urea and creatinine levels highlight the importance of considering the broader context when interpreting these biomarkers. In the context of our study, dogs in the control group may have exhibited slightly higher serum creatinine concentrations due to factors such as increased muscle mass or age-related variations, while the dogs with subclinical heart disease had slightly lower serum urea concentrations, possibly related to dietary differences or age-related factors.

Additionally, SDMA, a relatively modern indicator, has been incorporated into standard diagnostics, though its effectiveness is still debated. The inclusion of SDMA in renal assessments is a notable advancement in understanding kidney function, but its utility in specific populations, such as dogs with subclinical heart disease, requires further investigation [56,57,58,59]. In published studies, the authors indicate that there will be some biological and individual day-to-day variability with SDMA [60,61]. SDMA concentration may also be influenced by age, especially in very young dogs [59]. Further research is needed to determine the effect of breed on SDMA concentrations; however, Liffman et al. [62] found that greyhounds have significantly higher mean serum SDMA concentrations compared to dogs of other breeds. Additionally, some dogs with lymphoma had increased SDMA concentrations while remaining non-azotemic and showed a return to the normal value of SDMA after clinical remission after chemotherapy [63]. Due to the above limitations, this study did not measure SDMA concentration and focused on the search for other renal biomarkers.

Reduced kidney function, primarily filtration capacity, is detectable through routine laboratory tests, including serum creatinine and urea measurements. However, as previously discussed, these changes are influenced by various factors and are typically identified late, often coinciding with the onset of clinical symptoms. This limitation leaves the early phase of kidney disease largely undetected in routine laboratory evaluations. Therefore, the search for more sensitive and specific biomarkers, such as those investigated in this study, remains crucial for the early detection of kidney damage, especially in the context of subclinical heart disease in dogs.

To address this diagnostic gap, both human [64,65] and veterinary medicine [56,65,66] have proposed studying various indicators. Promising compounds under consideration include interleukin 18, liver-type fatty acid-binding protein (L-FABP), alpha-1 microglobulin, N-acetyl-beta-D-glucosaminidase (NAG), retinol-binding protein, uromodulin, as well as cystatin C, KIM-1, and NGAL. In this study, the authors focused on analyzing cystatin C, KIM-1, and NGAL measured in blood serum as biomarkers of early kidney damage in dogs with stage B2 DCM. Given the ambiguous results and interpretation challenges, an additional diagnostic factor was incorporated—the ratio of cystatin C, KIM-1, and NGAL concentration to creatinine level. This approach aims to enhance the effectiveness of measuring these biomarkers, especially during the early phase of kidney damage.

Cystatin C is recognized as a biomarker of proximal tubule damage due to its serum concentration primarily reflecting glomerular filtration, thus correlating significantly with the kidneys’ excretory capacity. In this study, the analysis of average cystatin C concentration and the cystatin C/creatinine ratio revealed marginally higher values in dogs with heart disease compared to the control group. Although these differences between dogs with DCM and the control group were slight, there was a trend toward higher results in dogs with subclinical heart disease.

The lack of differences in concentrations might be attributed to several factors. First, the study included dogs only at an early stage of heart disease, specifically stage B2 of DCM. Moreover, the group size was relatively small, which might have affected the statistical power of the study. It is possible that at this early stage of heart disease, there had not been a substantial reduction in glomerular filtration or significant damage to the tubular epithelium.

The cystatin C values obtained in healthy dogs in this study align with results reported by other authors [26,67,68]. Interestingly, there seems to be a lack of literature reports on cystatin C concentrations in the course of DCM, making these findings potentially valuable for future research and clinical practice. This study indicates that while cystatin C may be a useful biomarker for early renal damage in dogs with DCM, further research with larger groups and across different stages of heart disease is needed to confirm its diagnostic utility.

Kidney injury molecule-1 (KIM-1) is a cell membrane protein found in the proximal tubule epithelium of the nephron and is recognized as a biomarker of tubular damage. In human medicine, KIM-1 is established as an indicator of AKI and has also been proposed for use in the diagnosis of CKD. While there are some reports in the veterinary literature highlighting the utility of KIM-1 in assessing AKI in canine medicine, its application in the diagnosis of CKD in dogs remains less explored. However, studies conducted on rats suggest that KIM-1 might be valuable in tracking the progression and changes in CKD [14,15,68].

In our research, the average concentration of KIM-1 was found to be similar between dogs with DCM and the control group. The KIM-1/creatinine ratio in dogs with DCM was higher compared to healthy dogs, but these differences were not significant either. These findings suggest that in the early, asymptomatic stages of DCM, it is challenging to pinpoint the exact duration from the onset of myocardial changes to their echocardiographic detection. Consequently, evaluating and comparing the results of this study with others is difficult due to the scarcity of literature on KIM-1 concentration in dogs. The only available study referenced in this context involved AKI induced by gentamicin, where the findings by Zheng et al. [14] showed lower KIM-1 concentrations than those observed in the current study. The observed discrepancies might be attributed to differences in the ELISA tests used, which had varying detection and quantification ranges as per the manufacturers’ specifications. Moreover, the referenced study by Zheng et al. [14] involved only 16 dogs aged 12 to 16 months, limiting the breadth of comparative analysis. While KIM-1 shows promise as a biomarker for kidney damage in the context of DCM, more comprehensive research, including studies with larger sample sizes and across different stages of heart and kidney diseases, is needed to establish its diagnostic relevance in canine CKD.

Neutrophil gelatinase-associated lipocalin (NGAL) is a protein synthesized in various body tissues, and its production significantly increases during acute AKI and CKD. An elevated serum NGAL concentration is particularly indicative of renal tubular damage. As an acute-phase protein, NGAL plays a role in both acute and chronic inflammatory responses. Research in both human and veterinary medicine suggests that NGAL is a sensitive and specific biomarker, valuable for diagnosing early renal changes arising from diverse etiologies [69].

In dogs diagnosed with DCM, the study identified significant variations in serum levels of NGAL and the NGAL/creatinine ratio. This pattern suggests a likely reduction in glomerular filtration and damage to proximal renal tubules, potentially marking the onset of CKD related to heart disease. Similar observations were made in other studies. Cortellini et al. [70] noted elevated NGAL levels in sepsis cases, a finding echoed by Otto et al. [71]. Increased NGAL values in dogs with natural kidney diseases were reported by Hsu et al. [33]. Furthermore, Kim et al. [34] emphasized the diagnostic importance of NGAL measurement in urine for kidney disease, a perspective supported by Palm et al. [35] and Zheng et al. [14] in their studies on induced AKI. Steinbach et al. observed a similar trend, with normal NGAL values in healthy dogs but significantly higher levels in those with AKI and CKD [36].

Evaluating serum NGAL as a standalone biomarker appears to be effective for dogs with DCM, offering promising insights, particularly in light of the lack of veterinary literature on NGAL’s relationship with early-stage DCM in dogs. The value of the NGAL/creatinine ratio further supports this. Establishing such correlations underscores the potential of serum NGAL and the NGAL/creatinine ratio in identifying early kidney changes during DCM progression. However, further research involving more dogs is needed to validate these findings and to ascertain the usefulness of these biomarkers for monitoring the disease, especially regarding DCM’s impact on renal function.

## 5. Conclusions

This study’s findings indicate that during the asymptomatic phase of DCM, specifically at stage B2, serum concentrations of NGAL and the NGAL/creatinine ratio can serve as potential diagnostic markers for kidney damage. These findings, emerging from our research, could be instrumental as a preliminary basis for further studies in this area. Furthermore, they have the potential to stimulate discussions on the diagnostic significance of these biomarkers in identifying early renal changes in dogs. The 5 min ECG will also be replaced by a Holter recording for screening examination in Doberman Pinschers, Boxer dogs, and other breeds, like Great Danes, to distinguish, e.g., tachycardia-induced cardiomyopathy [7,23,72,73].

## Figures and Tables

**Table 1:** Summary of dog characteristics, selected hematology, and serum biochemistry. Data, except sex, are reported as mean values with standard deviations (SDs).

		Control (*n* = 17)	DCM (*n* = 9)	*p*-Value
		Mean ± SD	Mean ± SD	
		Dog Characteristics		
Sex	male/female	12/5	6/3	1
Age	years	5.50 ± 2.2	8.72 ± 3,55	<0.01
Body weight	kg	16.96 ± 10.1	33.9 ± 9.2	<0.01
BCS	9-point scale	4.59 ± 0.94	5.78 ± 1.85	0.04
Body temperature	°C	38.4 ± 0.4	38.5 ± 0.4	0.08
Heart rate	BPM	122 ± 18	128 ± 22	0.26
Systolic blood pressure	mmHg	134 ± 14	138 ± 11	0.67
		Hematology		
Packed cell volume	L/L	0.52 ± 0.08	0.49 ± 0.05	0.28
WBC	10^9^/L	7.06 ± 3.08	9.63 ± 3.54	0.08
		Serum Biochemistry		
Serum total protein	g/L	63 ± 3.8	65.2 ± 13	0.58
Urea	mmol/L	6.59 ± 2.38	7.3160 ± 1.6683	0.45
Creatinine	mmol/L	0.084 ± 0.015	0.0804 ± 0.017	0.54
AST	µkat/L	0.52 ± 0.14	0.65 ± 0.32	0.23
Na^+^	mmol/L	146.0 ± 4.83	142.65 ± 9.05	0.31
K^+^	mmol/L	4.56 ± 0.2	5.13 ± 1.42	0.11
Ca^2+^	mmol/L	1.39 ± 0.07	1.4 ± 0.01	0.26

BCS: body condition score; BPM: beats per minute; WBC: white blood cells; AST: aspartate transaminase; Na^+^: sodium; K^+^: potassium; Ca^2+^: ionized calcium.

**Table 2:** Summary of cystatin C, KIM-1, NGAL, cystatin C/creatinine ratio, KIM-1/creatinine, and NGAL/creatinine in serum at enrollment. Data are reported as mean values with standard deviations (SDs).

		Control (*n* = 17)	DCM (*n* = 9)	*p*-Value
		Mean ± SD	Mean ± SD	
Cystatin C	mg/L	0.24 ± 0.04	0.27 ± 0.1	0.32
Cystatin C/creatinine	ratio	0.26 ± 0.07	0.32 ± 0.12	0.45
KIM-1	ng/mL	0.61 ± 0.07	0.61 ± 0.08	0.91
KIM-1/creatinine	ratio	0.67 ± 0.13	0.69 ± 0.17	0.19
NGAL	ng/mL	2.76 ± 1.8	6.46 ± 5.22 A	0.02

## Data Availability

None of the data was deposited in an official repository. All the data obtained in the present research are presented in this manuscript. The data that support the study findings are available from the authors upon request.
